# Brazilian Foodborne Disease National Survey: Evaluating the Landscape after 11 Years of Implementation to Advance Research, Policy, and Practice in Public Health

**DOI:** 10.3390/nu11010040

**Published:** 2018-12-25

**Authors:** Cainara Lins Draeger, Rita de Cassia Coelho de Almeida Akutsu, Renata Puppin Zandonadi, Izabel Cristina Rodrigues da Silva, Raquel Braz Assunção Botelho, Wilma Maria Coelho Araújo

**Affiliations:** 1 Department of Nutrition, Faculty of Health Sciences, University of Brasilia, Brasilia 70910-900, Brazil; cainara@gmail.com (C.L.D.); rita.akutsu@gmail.com (R.d.C.C.d.A.A.); raquelbabotelho@gmail.com (R.B.A.B.); wilma.araujo@terra.com.br (W.M.C.A.); 2 Faculty of Ceilândia, University of Brasília, Brasília 72220-275, Brazil; belbiomedica@gmail.com

**Keywords:** national survey, foodborne disease, public health, health survey

## Abstract

The poor control of public and private agencies regarding the quality of foods offered to populations has a significant impact on the occurrence of foodborne diseases. Precise information about foodborne diseases (FBD) can adequately inform policy-makers and help to allocate appropriate resources for the control of food safety. This study aimed to evaluate the Brazilian foodborne disease landscape after 11 years of implementation of the Epidemiological Surveillance System of Foodborne Diseases. The study analyzed secondary data from the National System of Injuries and Notifications (SINAN-NET), available from the Health Department. We evaluated the characteristics of FBD, such as the food involved, the location of ingestion, the total time to the outcome investigation, the microorganism involved and deaths. We also calculated the global incidence, mortality and lethality rates of the country. There were 7630 FBD outbreaks in the National Epidemiological Surveillance System of Foodborne Diseases (VE-DTA). Of the registered reports, a total of 134,046 individuals were sick with FBD; 19,394 were hospitalized, and there were 127 registered deaths. We found a coefficient of incidence of FBD of 67.57 per 100,000 inhabitants; a mortality coefficient of 0.06 per 100,000 inhabitants and lethality of 0.09% over the 11 years investigated. Data are probably underreported since the VE-DTA system lacks completeness, and because FBD symptoms are mostly mild, a large part of the population does not seek care from health services.

## 1. Introduction

Foodborne diseases (FBD) are an important cause of morbidity and mortality, and they have been an issue for the worldwide population since the beginning of humanity. Their types and impacts have changed through the decades, yet they are still diverse across different communities and have an impact on public health [1,2,3]. The global burden of foodborne diseases is considerable, with regional variations evident. However, the full extent of unsafe food and its damage to the public has been unknown. Only a fraction of foodborne diseases is recognized, reported to public health authorities, and recorded in official disease statistics [2].

The increasing population growth, the existence of vulnerable population groups, the disorderly urbanization process, and the need for large-scale food production all contribute to the growing number of FBD [4]. The poor control of public and private agencies regarding the quality of foods offered to populations also has a significant impact the occurrence of FBD [5]. Despite the increasing international awareness of FBD as a significant risk to health and socioeconomic development, food safety is still marginalized. One of the main obstacles is the lack of data on the extent and the cost of foodborne diseases. Precise information about FBD can adequately inform policy-makers and help to allocate appropriate resources for food safety control and intervention efforts. Knowledge about FBD rates enables the promotion of actions at a national level, through national FBD studies encouraging the use of the information in public policies.

In this context, in 2007 the Brazilian Health Surveillance Secretariat of the Department of Health developed *the National Epidemiological Surveillance System of Foodborne Diseases* (*Sistema Nacional de Vigilância Epidemiológica das Doenças Transmitidas por Alimentos—VE-DTA*) [6]. This system aims to provide information to promote health actions, and reduce the incidence of FBD and of deaths caused by FBD in Brazil. Despite the efforts to record and investigate FBD in Brazil, there are still considerable difficulties in using data from the VE-DTA system, as evidenced by the lack of publications. The last FBD epidemiological bulletin is from the year 2005, and it uses data on FBD from 1994–2004 [7], before implementation of the VE-DTA system. Therefore, this study aimed to evaluate the Brazilian Foodborne disease landscape after 11 years of implementation of the *National Epidemiological Surveillance System of Foodborne Diseases*. This study should enable the Brazilian government and health stakeholders to draw public policy attention to this often underestimated problem and to mobilize political will and resources to prevent foodborne diseases, a significant public health issue.

## 2. Materials and Methods

This descriptive epidemiological study used historical data on foodborne diseases in Brazil, from 2007 to 2017 (that is, for the 11 years following implementation of the VE-DTA surveillance system). Secondary analysis of data from the National System of Injuries and Notifications (SINAN-NET), available from the Health Department, was undertaken. The system has a platform (VE-DTA) in which Brazilian FBD notifications are registered [5,6]. The use of secondary data from health information systems presents as advantages the broad population coverage, the low cost of collecting information and the facility for longitudinal follow-up [8].

We evaluated the characteristics of the FBD, such as the food involved, the location of ingestion, the total time to the outcome investigation, the microorganism involved and the number of deaths. We also calculated the global incidence, mortality and lethality rates of the country. We calculated the coefficient rate of the number of cases of FBD or the deaths for the national average population during the 11 years investigated, showing the results for every 100,000 inhabitants. We calculated the FBD lethality using the ratio between the number of deaths due to the FBD and the total number of patients in the period. We performed the statistical analyses using the SPSS program (version 22.0). The chi-square test was used to evaluate the association between categorical variables, with a confidence interval of 5% [9].

The VE-DTA system aims to provide information that promotes health actions to reduce the occurrence of FBD in the country [6]. VE-DTA information is nationwide, and is available on the internet, on request, for public download. The municipal secretariat must notify the state health secretariat about foodborne disease outbreaks. It is mandatory for local doctors and health professionals to inform the municipal state secretariat about these health events. The health professional must register notified and investigated foodborne outbreaks, including the place of occurrence, the number of people affected, the number of people hospitalized, the number of deaths; the etiologic agents, and the food involved [10].

### Ethical Aspects

The study used secondary data, respecting the confidentiality and anonymity of the subjects notified in the information systems. The research was registered in the National System of Ethics and Research (Sisnep)—protocol number 68211717.7.0000.0030 and approved by the Ethics and Research Committee of the University of Brasília (UnB)—number 2.366.390.

## 3. Results

This study provides the first national overview of Brazilian foodborne diseases (FBD) after 11 years of implementation of the *Epidemiological Surveillance System of Foodborne Diseases* (it began in 2007). Since the system was implemented, there have been 7630 FBD outbreaks recorded in the VE-DTA. Of the registered reports, a total of 134,046 individuals were sick with FBD, 19,394 were hospitalized, and 127 deaths were registered. We found a coefficient of incidence of FBD of 67.57 per 100,000 inhabitants, a mortality coefficient of 0.06 per 100,000 inhabitants, and lethality of 0.09% over the 11 years investigated.

Among the health problems related to food intake, those with the highest number of notifications related to intestinal infections (*n* = 6506, 85.3%), toxic effects of the use of unspecified substances (*n* = 557, 7.3%), and infectious diarrhea and gastroenteritis (*n* = 212, 2.8%).

**Figure 1:**
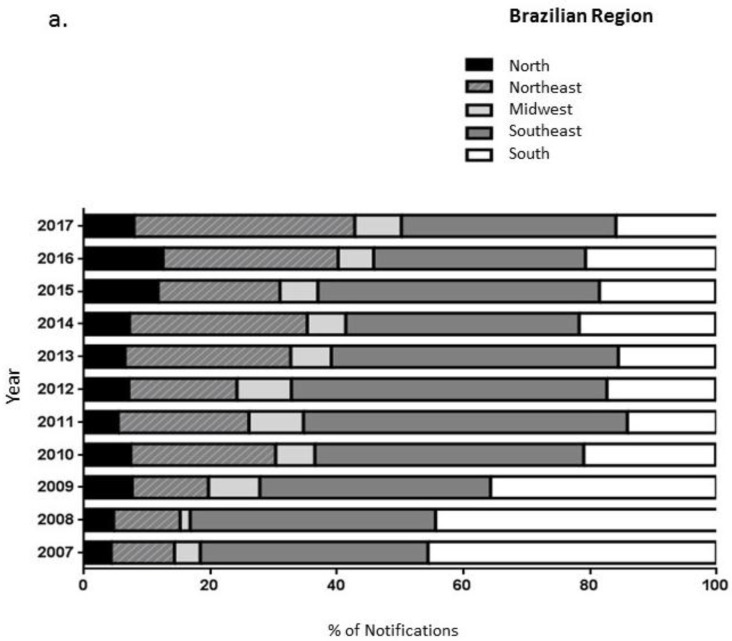

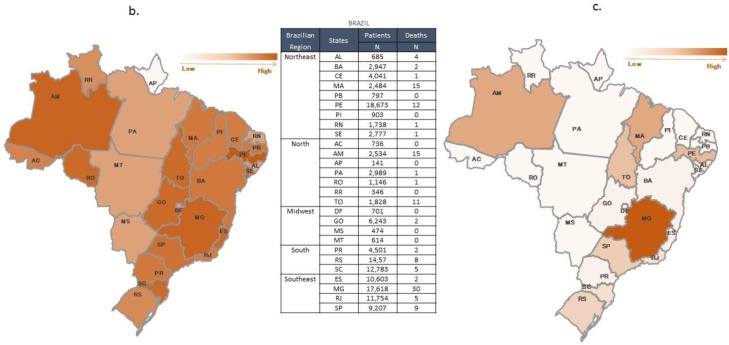
Foodborne disease outbreak notifications by Brazilian regions (**a**) and Brazilian states (**b**), and the number of deaths by Brazilian state (**c**). **Northeast Region-** AL: Alagoas, BA: Bahia, CE: Ceará, MA: Maranhão, PB: Paraíba, PE: Pernambuco, PI: Piauí, RN: Rio Grande do Norte, SE: Sergipe; **North Region-** AC: Acre, AM: Amazonas, AP: Amapá, PA: Pará, RO: Rondônia, RR: Roraima, TO: Tocantins; **Midwest Region-** DF: Distrito Federal, GO: Goiás, MT: Mato Grosso, MS: Mato Grosso do Sul; **South Region-** PR: Paraná, SC: Santa Catarina, RS: Rio Grande do Sul; **Southeast Region-** ES: Espírito Santo, MG: Minas Gerais, RJ: Rio de Janeiro, SP: São Paulo.

Analyzing the years of the notifications, proportionally, the year with the highest number of FBD outbreaks was 2014 (*n* = 886, 11.6%) and the year with the lowest was 2010 (*n* = 498, 6.5%) (Figure 1a). It is important to highlight that in 2014, “Fédération Internationale de Football Association” (FIFA) organized the world cup in Brazil. This event probably contributed to increase the number of FBD due to the number of people visiting Brazil and consequently increase in food production. As for the region of occurrence of the cases, we highlight the Southeast region with 3151 records (41.3%), followed by the South (*n* = 1824, 23.9%), Northeast (*n* = 1592, 120.9%), North (*n* = 556, 7.3%) and the Midwest (*n* = 482, 6.3%), respectively (Figure 1a). Evaluating the Brazilian states, we observed that Pernambuco, Minas Gerais, and the Rio Grande do Sul were the states with the highest FBD notifications; with 13.93%, 13.14%, and 10.87%, respectively (Figure 1b). Figure 1c demonstrates the number of deaths by state showing higher numbers of deaths in Minas Gerais (*n* = 30; Southeast region), followed by the states of Amazonas (*n* = 15) in the North, and Maranhão (*n* = 15) in the Northeast.

**Figure 2:**
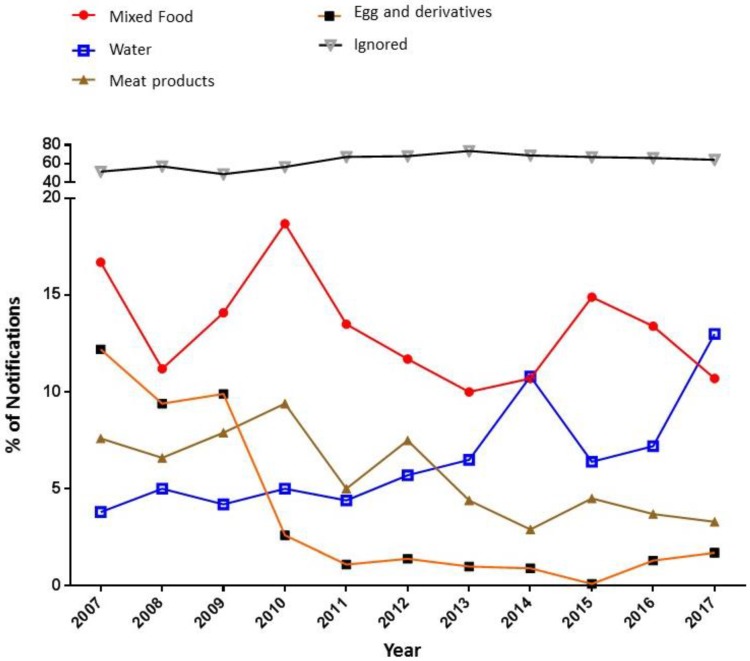
Percentage of notifications according to the food category between the years 2007 and 2017.

Regarding the local of outbreaks, most of the cases occurred in urban areas (83.1%), followed by rural areas (14.2%) with fewer occurrences in peripheral areas (2.3%). Unknown locality cases accounted for 0.5% of the total records. The analysis of the initial sites of outbreaks showed that the places with the highest occurrence were residences, with 2922 cases (38.3%). Restaurants and bakeries appeared in second place with 1265 cases (16.6%), followed by other sites such as lodges and workplaces, corresponding to 881 cases (11.5%). Considering the main foods that caused the outbreaks, unknown foods were most often registered for FBD cases (*n* = 4382, 57.4%), followed by mixed foods (*n* = 695, 9.1%), and water (*n* = 504, 6.6%) (Figure 2). There was also a statistically significant difference between the consumption of mixed foods (x² = 43.302, gl = 10, *p* = 0.000) and water (x² = 92,691, gl = 10, *p* = 0.000) regarding the year of FBD occurrence. It is noteworthy that there was an increase between the years 2016 and 2017 in the number of cases of food outbreaks caused by consumption of contaminated water, rising from 7.2% in 2016 to 13.0% in 2017 (Figure 2).

**Figure 3:**
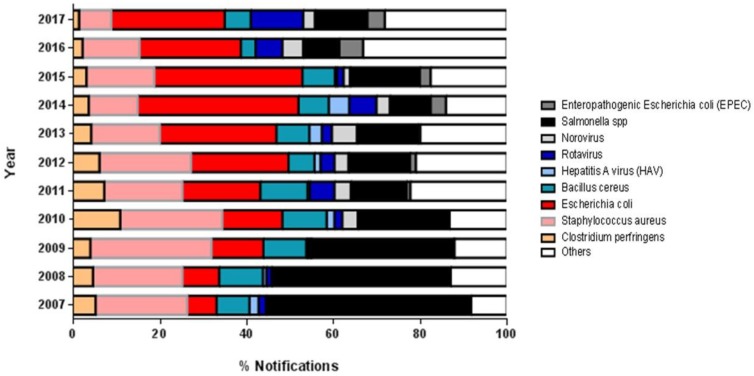
Percentage of notifications according to the etiological agent of the foodborne disease (2007 to 2017).

Regarding the etiological agent, the most involved microorganisms were *Salmonella* spp. (22.1% of all cases), followed by *Escherichia coli* (20.5%) and *Staphylococcus aureus* (18.2%) (Figure 3). Figure 3 presents the microorganisms that caused FBD at a frequency higher than 0.4% per year. We included those with a frequency lower than 0.4% in the “others” category. 

Evaluating the main etiological agents involved in the FBD outbreaks over the years, we found tendencies towards a decrease in the occurrence of outbreaks caused by *Salmonella* spp. and an increase in the number of cases of infection by *E. coli* (Figure 3).

Among the most used confirmatory criteria for outbreak investigations, the epidemiological clinical examination was the most frequent, with 38.7% of all cases, followed by inconclusive analysis, with a total of 20.3% of cases. The clinical laboratory criterion showed a frequency of 14.1%, bromatological laboratory 10.1%, and laboratory clinical bromatological 5.9%, with a significant difference (x² = 246.8, gl = 40, *p* < 0.0001). In general, we did not observe a great variation during the 11 years.

Regarding the states with the highest number of FBD records (Pernambuco, Minas Gerais, and the Rio Grande do Sul), all had unknown foods as the main cause of the outbreaks (40.4%, 68.6%, and 55%, respectively). For the place of consumption of food, the highest incidence of FBD was with food consumed in residences (34.5%, 42.8%, and 41.8%, respectively), and microbiological analyses with an unknown etiological agent (21.4%, 71%, and 55.6%, respectively). From the data recorded in the state of Amazonas, we observed that 86.6% of cases (*n* = 13) were attributed to contamination with the bacteria *Salmonella* spp. and 100% of cases were associated with food consumption within households. In 86.6% (*n* = 13) of the deaths, the food causing the outbreak of FBD was unknown. In Maranhão, it was observed that 100% (*n* = 15) of the deaths were caused by rotavirus infection after the consumption of contaminated water. Finally, in Minas Gerais, one-third (*n* = 10) of the deaths from FBD occurred in rest homes involving, for the most part, the consumption of unknown foods (*n* = 19). Regarding the tests applied to identify the etiological agent causing the outbreak, 53.3% (*n* = 16) had an unknown result.

We found that, on average, the time required for the investigation of outbreaks in Brazil was 54 days (53.4 ± 66.3 days). With the exception of 2007, there was a tendency for the investigation time of the cases to decrease over the years (Figure S1).

## 4. Discussion

Foodborne diseases represent one of the most common and important public health problems in the world, especially in developing countries that still have serious shortcomings in infrastructure and basic sanitation. In general, the countries that present the highest occurrence rates are those that have the least resources to prevent them [2,5]. Data from Canada’s health agency show that in 2016, 1.6 million people became ill, 4000 were hospitalized and 105 died from FBD in that country [11]. According to the World Health Organization (WHO) [2], 23 million people in Europe become ill and 5000 die from FBD every year.

The Canadian and the European epidemiological data regarding FBD are very different from the data found in Brazil in the present study. The data from Canada and Europe refer to a one year period of investigation, whereas in Brazil from 2007 to 2017, 134,046 cases of foodborne disease were registered, this being an extremely low number for an 11-year period. It is possible that the result found was due to the low level of completeness of the Brazilian National Epidemiological Surveillance System of Foodborne Diseases (VE-DTA), as discussed in a previous study [12].

It is important to consider that the national and international reports show that most cases of FBD are not notified to health authorities; because many foodborne pathogens cause mild symptoms, victims do not seek medical help, contributing to underreporting of epidemiological data. The number of reported cases can be considered the tip of the iceberg, compared with the actual number of cases [13].

Evaluating the Brazilian regions, the findings showed that the Southeast and South regions present larger proportions than the other regions of Brazil. This can be justified by the greater population density of these regions, corresponding with the higher number of reports of FBD [14]. It is also important to mention that in the Southeast region, the VE-DTA system presents the highest level of completeness when compared to other Brazilian regions[12]. Also, we observed that most outbreak reports were for those that occurred in urban areas (83.1%) compared to rural and peripheral areas. This fact is probably due, once again, to the greater population density of urban areas and the greater coverage of VE-DTA in these areas. According to the national survey conducted in Brazil, 84.72% of the population live in urban areas, and 15.28% live in rural areas [14]. We also observed that the rural population had less access to health services, less coverage by health plans and worse health conditions [15]. The lower access to health services contributes directly to the low notification of FBD found in these areas. Therefore, the results do not necessarily reflect a higher risk of FBD in rural and peripheral areas.

The occurrence of FBD outbreaks in residences shows a serious problem regarding the lack of sanitary education and knowledge about adequate preparation and storage of food by the population in general [16]. Outbreaks of foodborne diseases in households tend to be less well-known because they involve a smaller number of people (usually family). This fact contributes to the lack of direction in educational campaigns and training for this public [17].

We observed that the “unknown foods” group caused 57.4% of the FBD outbreaks. Since, in these instances, people do not know which food caused the FBD, it makes it difficult to investigate the outbreak. The state with the highest number of deaths per FBD in the country was Minas Gerais (*n* = 30), and the Amazonas state also recorded a high percentage of cases associated with the consumption of unknown foods (63.3%, *n* = 19 and 86.6%, *n* = 13, respectively). Also, the etiological agents have different incubation times, making it even more difficult to associate FBD symptoms with the food consumed and the etiological agent involved [18]

The second group of foods with a higher occurrence of FBD was mixed foods. These foods presented various ingredients in their composition and were usually homemade, which favors contamination and, consequently, transmission of FBD agents to consumers [5].

Another component that has a significant role in the occurrence of FBD outbreaks is water. Its contamination is directly related to the precariousness of the water supply network and water treatment. According to data from the World Health Organization (WHO) [19], contaminated water is related to most of the health and disease problems of the population worldwide. According to the latest report on water quality, 2.1 billion people worldwide do not have access to safe water at home, and 4.5 billion people lack safe sanitation [19].

In this study, there was an increase in the number of cases of waterborne diseases between 2016 and 2017. This fact can be observed especially in the state of Maranhão (Northeast region) where 100% of the deaths (*n* = 15) were due to the consumption of contaminated water. According to the Brazilian National Water Agency, 2016 was the most critical year for the impacts of the national water crisis on the population. Almost 18 million people were affected by climate phenomena that caused water scarcity. Difficulties accessing water lead to the consumption of poor quality water and an increase in the occurrence of waterborne and foodborne diseases since the water is also used to prepare food [20].

Regarding the etiological agents most involved in FBD outbreaks in Brazil, there are more cases of food contaminated with *Salmonella* spp. (22.1%), *Escherichia coli* (20.5%), and *Staphylococcus aureus* (18.2%). However, we found that in most of the registered outbreaks the etiological agents were not identified, hindering the process of FBD investigation. This compares to Canada, where according to the Canadian Food Inspection Agency and Health Canada, the most involved agents were norovirus (responsible for 65% of FBD) followed by *Clostridium perfringens* (11%), *Campylobacter* spp. (8%) and *Salmonella* spp. (5%) [21]. In Europe, the most involved etiologic agents were norovirus (15 million cases) and *Campylobacter* (approximately 5 million cases) [22]. In the United States (US), the top five FBD etiological agents are norovirus, *Salmonella*, *Clostridium perfringen*s, *Campylobacter*, and *Staphylococcus aureus*, respectively [18].

That the highest incidence of outbreaks involving norovirus are in Canada, the US, and Europe is expected since the noroviruses are common in North America and Europe and are very contagious, affecting all age groups. Norovirus illness can happen year-round, but outbreaks are more common in the fall and winter months [21,23]. Brazil, because of its warmer climate, has a slightly different profile of etiological agents. Higher temperatures have the most noticeable effect on salmonellosis reports. However, the potential risks of high temperatures related to tropical climates can be neutralized through public health actions. Thus, regardless of climatic factors, health interventions, education, and food safety standards should be able to mitigate the possible negative consequences of the occurrence of FBD [24,25].

According to the WHO, the geographic location of some countries contributes to the occurrence of FBD. Pests, microorganisms, and the formation of toxins are more likely in countries with tropical climates. At the same time, many of these countries are still developing and have no production and storage control measures to ensure complete food safety. A large part of the world’s population is both economically disadvantaged and suffering from recurrent FBD, which aggravate their (often already weakened) health situations; in turn, contributing to delayed physical and mental development and depriving individuals of opportunities to achieve their full development and work potential in society. The FBD recurrence picture in developing countries leads to a vicious cycle that weakens the poorest population and leads to the loss of social mobility capacity [2].

The confirmation criteria used by the health services to investigate the etiological agent underlying a foodborne disease outbreak are based on the following five types of investigations: (1) the clinical laboratory criterion, when the cause of the outbreak is concluded based only on the results of the clinical samples; (2) the clinical epidemiological criterion, which is used when there are no clinical samples collected, when the laboratory results are negative, or when the laboratory results are not compatible with the clinical presentation and epidemiology of the outbreak; (3) bacteriological laboratory criteria, when the cause of the outbreak is concluded based only on the results of the bromatological samples; (4) clinical and bromatological laboratory criteria, when the cause of the outbreak is concluded based on the results of clinical and bromatological samples—that is, when the same etiological agent is identified in the clinical and bromatological samples; and finally (5) the inconclusive criterion, which is used when there is no information that allows the cause of the outbreak to be determined using the previous criteria [5].

The results of the present study showed that the most used confirmatory criteria were the clinical epidemiological and the inconclusive criteria, a fact which is considered unhelpful for clarifying cases of FBD. As can be seen from the above description, the epidemiological and inconclusive clinical criteria are used when the other criteria are not available or when there is insufficient information to resolve the case by another criterion. This demonstrates the difficulty of investigating FBD outbreaks, since often health professionals do not have the information they need to conduct a full investigation of the case.

Regarding the average time taken for investigation of the outbreaks, there was a decreasing trend after 2008 and 2009. We can justify this trend since the VE-DTA system started in 2007 and the following two years were probably years of adaptation, [12]. Thus, we observed that there was an improvement in the mean time taken for investigation over the 11 years [5].

Most FBD outbreaks could be avoided if the food production chain adopted preventive behaviors. The Codex Alimentarius, created in 1963, is a collection of internationally adopted food standards aimed at protecting the health of consumers and ensuring fair practices in regional and international food trade. The Codex Alimentarius recommendations are voluntary but, in most cases, they are the basis for the creation of national food production standards [26].

In Brazil, the National Agency of Sanitary Surveillance (ANVISA) carries out the inspection and maintenance of legislation related to sanitation and sanitary control and good manufacturing practices for food-producing establishments. By Ordinance No. 326 of July 30, 1997, an appropriate methodology for assessing the risks of contamination of food in the various stages of production contained in the Regulation should be used, together with intervening whenever necessary to ensure food is fit for human consumption.

Given Brazil’s continental dimensions, an inspection action that covers all food producing establishments becomes complex, despite the existing legislation and the control measures already implemented. Thus, the country still faces problems in the control of FBD throughout its territory, as evidenced in the data presented in this work.

## 5. Conclusions

The present study found that the occurrence of FBD in Brazil is low when compared to other countries. However, the data are probably underreported since the VE-DTA system lacks completeness, and because FBD symptoms are mostly mild, a large part of the population does not seek health care. The country still faces problems in controlling FBD across its territory. In this sense, we recommend that there is a greater focus on educational activities and preventive actions related to food handling and storage with the entire population, since most food-related outbreaks originate in a residential environment.

## Supplementary Materials

The following are available online at http://www.mdpi.com/2072-6643/11/1/40/s1, Figure S1: Mean time and standard deviation of outbreak investigation between the years 2007 and 2017.
