# Supplementary figures and images for: Brazilian Foodborne Disease National Survey: Evaluating the Landscape after 11 Years of Implementation to Advance Research, Policy, and Practice in Public Health
Source: Nutrients. 2018 Dec 25;11(1):40. doi: 10.3390/nu11010040 (PMC6356215; doi:10.3390/nu11010040)

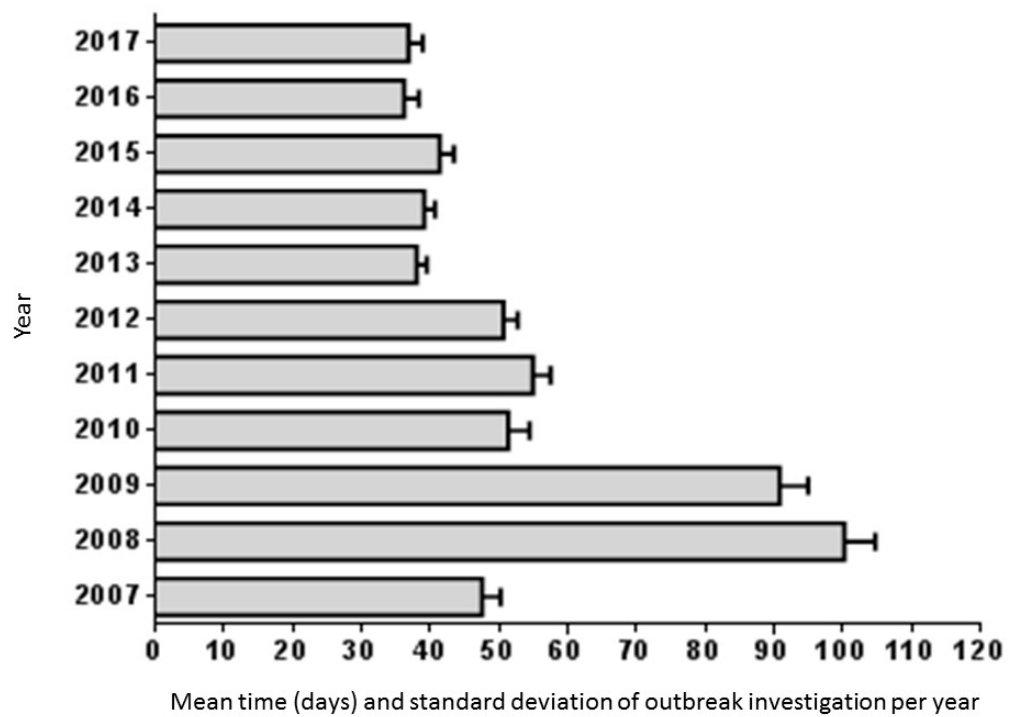

Figure S1: Mean time and standard deviation of outbreak investigation between the years 2007 and 2017

Supplement: Supplementary file 1 [file nutrients-11-00040-s001.pdf]
